# A tutorial on multiobjective optimization: fundamentals and evolutionary methods

**DOI:** 10.1007/s11047-018-9685-y

**Published:** 2018-05-31

**Authors:** Michael T. M. Emmerich, André H. Deutz

**Affiliations:** LIACS, Leiden University, Leiden, The Netherlands

**Keywords:** Multiobjective optimization, Multiobjective evolutionary algorithms, Decomposition-based MOEAs, Indicator-based MOEAs, Pareto-based MOEAs, Performance assessment

## Abstract

In almost no other field of computer science, the idea of using bio-inspired search paradigms has been so useful as in solving multiobjective optimization problems. The idea of using a population of search agents that collectively approximate the Pareto front resonates well with processes in natural evolution, immune systems, and swarm intelligence. Methods such as NSGA-II, SPEA2, SMS-EMOA, MOPSO, and MOEA/D became standard solvers when it comes to solving multiobjective optimization problems. This tutorial will review some of the most important fundamentals in multiobjective optimization and then introduce representative algorithms, illustrate their working principles, and discuss their application scope. In addition, the tutorial will discuss statistical performance assessment. Finally, it highlights recent important trends and closely related research fields. The tutorial is intended for readers, who want to acquire basic knowledge on the mathematical foundations of multiobjective optimization and state-of-the-art methods in evolutionary multiobjective optimization. The aim is to provide a starting point for researching in this active area, and it should also help the advanced reader to identify open research topics.

## Introduction

Consider making investment choices for an industrial process. On the one hand the profit should be maximized and on the other hand environmental emissions should be minimized. Another goal is to improve safety and quality of life of employees. Even in the light of mere economical decision making, just following the legal constraints and minimizing production costs can take a turn for the worse.

Another application of multiobjective optimization can be found in the medical field. When searching for new therapeutic drugs, obviously the potency of the drug is to be maximized. But also the minimization of synthesis costs and the minimization of unwanted side effects are much-needed objectives (van der Horst et al. 2012; Rosenthal and Borschbach 2017).

There are countless other examples where multiobjective optimization has been applied or is recently considered as a promising field of study. Think, for instance, of the minimization of different types of error rates in machine learning (false positives, false negatives) (Yevseyeva et al. 2013; Wang et al. 2015), the optimization of delivery costs and inventory costs in logistics (Geiger and Sevaux 2011), the optimization of building designs with respect to health, energy efficiency, and cost criteria (Hopfe et al. 2012).

In the following, we consider a scenario where given the solutions in some space of possible solutions, the so-called *decision space* which can be evaluated using the so-called *objective functions*. These are typically based on computable equations but might also be the results of physical experiments. Ultimately, the goal is to find a solution on which the decision maker can agree, and that is optimal in some sense.

When searching for such solutions, it can be interesting to pre-compute or approximate a set of interesting solutions that reveal the essential trade-offs between the objectives. This strategy implies to avoid so-called *Pareto dominated solutions*, that is solutions that can improve in one objective without deteriorating the performance in any other objective. The Pareto dominance is named after Vilfredo Pareto, an Italian economist. As it was earlier mentioned by Francis Y.Edgeworth, it is also sometimes called Edgeworth-Pareto dominance (see Ehrgott 2012 for some historical background). To find or to approximate the set of non-dominated solutions and make a selection among them is the main topic of multiobjective optimization and multi-criterion decision making. Moreover, in case the set of non-dominated solutions is known in advance, to aid the decision maker in selecting solutions from this set is the realm of decision analysis (aka decision aiding) which is also part of multi-criterion decision making.

### **Definition 1**

Multiobjective Optimization. Given *m* objective functions $f_1: {{\mathcal {X}}} \rightarrow {\mathbb {R}}, \dots , f_m: {{\mathcal {X}}} \rightarrow {\mathbb {R}}$ which map a decision space ${\mathcal {X}}$ into ${\mathbb {R}}$, a multiobjective optimization problem (MOP) is given by the following problem statement:

1 $$\begin{aligned} \text{ minimize } f_1({\mathbf {x}}) , \dots , \text{ minimize } f_m({\mathbf {x}}), {\mathbf {x}} \in {\mathcal {X}} \end{aligned}$$

### *Remark 1*

In general, we would demand $m>1$ when we talk about multiobjective optimization problems. Moreover, there is the convention to call problems with large *m*, not multiobjective optimization problems but *many-objective optimization problems* (see Fleming et al. 2005; Li et al. 2015). The latter problems form a special, albeit important case of multiobjective optimization problems.

### *Remark 2*

Definition 1 does not explicitly state constraint functions. However, in practical applications constraints have to be handled. Mathematical programming techniques often use linear or quadratic approximations of the feasible space to deal with constraints, whereas in evolutionary multiobjective optimization constraints are handled by penalties that increase the objective function values in proportion to the constraint violation. Typically, penalized objective function values are always higher than objective function values of feasible solutions. As it distracts the attention from particular techniques in MOP solving, we will only consider unconstrained problems. For strategies to handle constraints, see Coello Coello (2013).

Considering the point(s) in time when the decision maker interacts or provides additional preference information, one distinguishes three general approaches to multiobjective optimization (Miettinen 2012):

1. A priori: A total order is defined on the objective space, for instance by defining a utility function ${\mathbb {R}}^m \rightarrow {\mathbb {R}}$ and the optimization algorithm finds a minimal point (that is a point in ${\mathcal {X}}$) and minimum value concerning this order. The decision maker has to state additional preferences, e.g., weights of the objectives, *prior* to the optimization.
2. A posteriori: A partial order is defined on the objective space ${\mathbb {R}}^m$, typically the Pareto order, and the algorithm searches for the minimal set concerning this partial order over the set of all feasible solutions. The user has to state his/her preferences *a posteriori*, that is after being informed about the trade-offs among non-dominated solutions.
3. Interactive (aka Progressive): The objective functions and constraints and their prioritization are refined by requesting user feedback on preferences at multiple points in time during the execution of an algorithm.

In the sequel, the focus will be on a posteriori approaches to multiobjective optimization. The a priori approach is often supported by classical single-objective optimization algorithms, and we refer to the large body of the literature that exists for such methods. The a posteriori approach, however, requires interesting modifications of theorems and optimization algorithms—in essence due to the use of partial orders and the desire to compute a set of solutions rather than a single solution. Interactive methods are highly interesting in real-world applications, but they typically rely upon algorithmic techniques used in a priori and a posteriori approaches and combine them with intermediate steps of preference elicitation. We will discuss this topic briefly at the end of the tutorial.

## Related work

There is a multiple of introductory articles that preceded this tutorial:

- In Zitzler et al. (2004) a tutorial on state-of-the-art evolutionary computation methods in 2004 is provided including Strength Pareto Evolutionary Algorithm Version 2 (SPEA2) (Zitzler et al. 2001), Non-dominated Sorting Genetic Algorithm II (NSGA-II) (Deb et al. 2002), Multiobjective Genetic Algorithm (MOGA) (Fonseca and Fleming 1993) and Pareto-Archived Evolution Strategy (PAES) (Knowles and Corne 2000) method. Indicator-based methods and modern variants of decomposition based methods, that our tutorial includes, were not available at that time.
- In Deb (2008) an introduction to earlier multiobjective optimization methods is provided, and also in the form of a tutorial. The article contains references to early books in this field and key articles and also discusses applications.
- Derivative-free methods for multiobjective optimization, including evolutionary and direct search methods, are discussed in Custódio et al. (2012).
- On conferences such as GECCO, PPSN, and EMO there have been regularly tutorials and for some of these slides are available. A very extensive tutorial based on slides is the citable tutorial by Brockhoff (2017).

Our tutorial is based on teaching material and a reader for a course on Multiobjective Optimization and Decision Analysis at Leiden University, The Netherlands (http://moda.liacs.nl). Besides going into details of algorithm design methodology, it also discusses foundations of multiobjective optimization and order theory. In the light of recent developments on hybrid algorithms and links to computational geometry, we considered it valuable to not only cover evolutionary methods but also include the basic principles from deterministic multiobjective optimization and scalarization-based methods in our tutorial.

## Order and dominance

For the notions we discuss in this section a good reference is Ehrgott (2005).

The concept of Pareto dominance is of fundamental importance to multiobjective optimization, as it allows to compare two objective vectors in a precise sense. That is, they can be compared without adding any additional preference information to the problem definition as stated in Definition 1.

In this section, we first discuss partial orders, pre-orders, and cones. For partial orders on ${\mathbb {R}}^m$ there is an important geometric way of specifying them with cones. We will define the Pareto order (aka Edgeworth-Pareto order) on ${\mathbb {R}}^m$. The concept of Pareto dominance is of fundamental importance for multiobjective optimization, as it allows to compare two objective vectors in a precise sense (see Definition 5 below). That is, comparisons do not require adding any additional preference information to the problem definition as stated in Definition 1. This way of comparison establishes a pre-order (to be defined below) on the set of possible solutions (i.e., the decision space), and it is possible to search for the set of its minimal elements—the efficient set.

As partial orders and pre-orders are special binary relations, we digress with a discussion on binary relations, orders, and pre-orders.

### **Definition 2**

Properties of Binary Relations. Given a set *X*, a binary relation on *X*—that is a set *R* with $R \subseteq X \times X$—is said to be

- reflexive, if and only if $\forall x \in X: (x,x) \in R$,
- irreflexive, if and only if $\forall x \in X, (x,x) \not \in R$,
- symmetric, if and only if $\forall x\in X: \forall y\in X: (x,y) \in R \Leftrightarrow (y,x) \in R$,
- asymmetric, if and only if $\forall x\in X: \forall y\in X: (x,y) \in R \Rightarrow (y,x) \not \in R$,
- antisymmetric, if and only if $\forall x\in X: \forall y\in X: (x,y) \in R \wedge (y,x) \in R \Rightarrow x=y$,
- transitive, if and only if $\forall x\in X: \forall y\in X: \forall z \in X: (x,y)\in R \wedge (y,z)\in R \Rightarrow (x, z)\in R$.

### *Remark 3*

Sometimes we will also write *xRy* for $(x,y) \in R$.

Now we can define different types of orders:

### **Definition 3**

Pre-order, Partial Order, Strict Partial Order. A binary relation *R* is said to be a

- pre-order (aka quasi-order), if and only if it is transitive and reflexive,
- partial order, if and only if it is an antisymmetric pre-order,
- strict partial order, if and only if it is irreflexive and transitive

### *Remark 4*

Note that a strict partial order is necessarily asymmetric (and therefore also anti-symmetric).

### Proposition 1

*Let* *X* *be a set and* $\Delta = \{ (x, x) | x \in X \}$ *be the diagonal of* *X*.

- *If R* *is an anti-symmetric binary relation on* *X*, *then any subset of R* *is also an anti-symmetric binary relation.*
- *If R* *is irreflexive, then* (*R* *is asymmetric if and only if* *R* *is antisymmetric*). *Or: the relation* *R* *is asymmetric if and only if* *R* *is anti-symmetric and irreflexive.*
- *If* *R* *is a pre-order on* *X*, *then* $\{ (x,y)\, |\, (x,y) \in R \text{ and } (y,x) \not \in R \}$, *denoted by* $R_{\text{ strict }}$, *is transitive and irreflexive. In other words,* $R_{\text{ strict }}$ *is a strict partial order associated to the pre-order* *R*.
- *If* *R* *is a partial order on* *X*, *then* $R \setminus \Delta$ *is irreflexive and transitive. In other words,* $R \setminus \Delta$ *is a strict partial order. Moreover* $R \setminus \Delta$ *is anti-symmetric (or asymmetric)*.
- *If R* *is a pre-order on* *X*, *then* ($R \setminus \Delta$ *is a strict partial order if and only if* *R* *is asymmetric*).

### *Remark 5*

In general, if *R* is a pre-order, then $R \setminus \Delta$ does not have to be transitive. Therefore, in general, $R \setminus \Delta$ will not be a strict partial order.

### **Definition 4**

Minimal Element. A minimal element $x\in X$ in a (strictly) partially ordered set (*X*, *R*) is an element for which there does not exist an $x' \in X$ with $x' R x$ and $x'\ne x$. (In case, the order *R* is a strict partial order, $x' R x$ implies $x'\ne x$).

### **Definition 5**

Pareto Dominance. Given two vectors in the objective space, that is ${\mathbf {y}}^{(1)}\in {\mathbb {R}}^m$ and ${\mathbf {y}}^{(2)}\in {\mathbb {R}}^m$, then the point ${\mathbf {y}}^{(1)} \in {\mathbb {R}}^m$ is said to *Pareto dominate* the point ${\mathbf {y}}^{(2)}$ (in symbols ${\mathbf {y}}^{(1)} \prec _{Pareto} {\mathbf {y}}^{(2)})$, if and only if

$$\begin{aligned} \forall i \in \{1, \dots , m \}: y_i^{(1)} \le y_i^{(2)} \text{ and } \exists j \in \{1, \dots , m \}: y_j^{(1)} < y_j^{(2)}. \end{aligned}$$

In words, in case that ${\mathbf {y}}^{(1)} \prec _{Pareto} {\mathbf {y}}^{(2)}$ the first vector is not worse in each of the objectives and better in at least one objective than the second vector.

### Proposition 2

*The Pareto order* $\prec _{Pareto}$ *on the objective space* ${\mathbb {R}}^m$ *is a strict partial order. Moreover* $(\prec _{Pareto} \cup \, \Delta )$ *is a partial order. We denote this by* $\preceq _{Pareto}$ *or also by* $\preceq$ *if the context provides enough clarity.*

In multiobjective optimization we have to deal with two spaces: The *decision space*, which comprises all candidate solutions, and the *objective space* which is identical to ${\mathbb {R}}^m$ and it is the space in which the objective function vectors are represented. The vector-valued function ${\mathbf {f}} = (f_1, \dots , f_m)^{\top }$ maps the decision space ${\mathcal {X}}$ to the objective space ${\mathbb {R}}^m$. This mapping and the Pareto order on ${\mathbb {R}}^m$ as defined in Definition 5 can be used to define a pre-order on the decision space ${\mathcal {X}}$ as follows.

### **Definition 6**

Pre-order on Search Space. Let $x_1, x_2 \in {\mathcal {X}}$. The solution $x_1$ is said to Pareto dominate the solution $x_2$ if and only if ${\mathbf {f}}(x_1) \prec _{Pareto} {\mathbf {f}}(x_2)$. Notation: $x_1$ Pareto dominates $x_2$ is denoted by $x_1 \prec _{{\mathbf {f}}} x_2$.

### *Remark 6*

The binary relation $\prec _{{\mathbf {f}}}$ on ${\mathcal {X}}$ is a strict partial order on ${\mathcal {X}}$ and $(\prec _{{\mathbf {f}}} \cup \{ (x,x)\, |\, x \in {\mathcal {X}} \})$ is a partial order on ${\mathcal {X}}$. Note that the pre-order *R* associated to $\preceq _{\text{ Pareto }}$ via ${\mathbf {f}}$ (  i.e., $x_1 R x_2$ if and only if ${\mathbf {f}}(x_1) \preceq _{\text{ Pareto }} {\mathbf {f}}(x_2)$  ) is, in general, not asymmetric and therefore, in general, $\prec _{{\mathbf {f}}}\, \not =\, R\, \setminus \, \{ (x,x)\, |\, x \in {\mathcal {X}} \}$.

Sometimes we need the notion of the so called strict component order on ${\mathbb {R}}^m$ and its accompanying notion of weak non-dominance.

### **Definition 7**

Strict Component Order on ${\mathbb {R}}^m$. Let ${\mathbf {x}}, {\mathbf {y}} \in {\mathbb {R}}^m$. We say ${\mathbf {x}}$ is less than ${\mathbf {y}}$ in the strict component order, denoted by ${\mathbf {x}} < {\mathbf {y}}$, if and only if $x_i < y_i, i=1,\dots , m$.

### **Definition 8**

(Weakly) Efficient Point, Efficient Set, and Pareto Front.

- The minimal elements of the Pareto order $\prec _{{\mathbf {f}}}$ on ${\mathcal {X}}$ are called *efficient* points.
- The subset ${\mathcal {X}}_E$ of all efficient points in ${\mathcal {X}}$ is called the *efficient set*.
- Let us denote the set of attainable objective vectors with ${\mathcal {Y}}:= {\mathbf {f}}({\mathcal {X}})$. Then the minimal elements of the Pareto order on ${\mathcal {Y}}$ are called the non-dominated or Pareto optimal objective vectors. The subset of all non-dominated objective vectors in ${\mathcal {Y}}$ is called the *Pareto front*. We denote it with ${\mathcal {Y}}_N$.
- A point $x \in {\mathcal {X}}$ is called weakly efficient if and only if there does not exist $u \in {\mathcal {X}}$ such that ${\mathbf {f}}(u) < {\mathbf {f}}(x)$. Moreover, ${\mathbf {f}}(x)$ is called weakly non-dominated.

### *Remark 7*

Clearly, $${{\mathbf {f}}}({\mathcal {X}}_E) = {\mathcal {Y}}_N$$.

### Cone orders

The Pareto order is a special case of a cone order, which are orders defined on vector spaces. Defining the Pareto order as a cone order gives rise to geometrical interpretations. We will introduce definitions for ${\mathbb {R}}^m$, although cones can be defined on more general vector spaces, too. The binary relations in this subsection are subsets of ${\mathbb {R}}^m \times {\mathbb {R}}^m$ and the cones are subsets of ${\mathbb {R}}^m$.

#### **Definition 9**

Non-trivial Cone. A set ${\mathcal {C}} \subset {\mathbb {R}}^m$ with $\emptyset \ne {\mathcal {C}} \ne {\mathbb {R}}^m$ is called a non-trivial cone, if and only if $\forall \alpha \in {\mathbb {R}}, \alpha >0, \forall c \in {\mathcal {C}}: \alpha c \in {\mathcal {C}}$.

#### *Remark 8*

In the sequel when we say cone we mean non-trivial cone.

#### **Definition 10**

Minkowski Sum. The Minkowski sum (aka algebraic sum) of two sets $A \in {\mathbb {R}}^m$ and $B\in {\mathbb {R}}^m$ is defined as $A \oplus B := \{ a + b\, |\, a\in A \wedge b\in B\}$. Moreover we define $\alpha A = \{\alpha a |\, a\in A\}$.

#### *Remark 9*

**Fig. 1:**
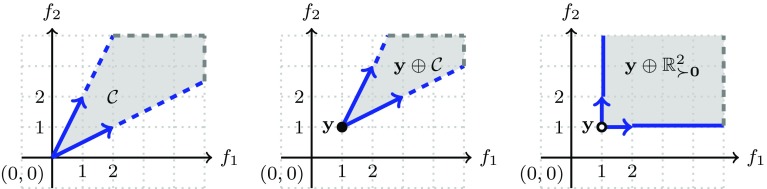
Example of a cone ${\mathcal {C}}$(left), Minkowski sum of a singleton $\{{\mathbf {y}}\}$ and ${\mathcal {C}}$ (middle), and Minkowski sum of $\{{\mathbf {y}}\}$ and the cone ${\mathbb {R}}^2_{\succ {\mathbf {0}}}$. The latter is equal to the non-negative quadrant from which the origin is deleted, see also Definition 13

For an illustration of the cone notion and examples of Minkowski sums see Fig. 1.

#### **Definition 11**

The binary relation, $R_{{\mathcal {C}}}$, associated to the cone ${\mathcal {C}}$. Given a cone ${\mathcal {C}}$ the binary relation associated to this cone, notation $R_{{\mathcal {C}}}$, is defined as follows: $\forall {\mathbf {x}} \in {\mathbb {R}}^m: \forall {\mathbf {y}} \in {\mathbb {R}}^m: ({\mathbf {x}}, {\mathbf {y}}) \in R_{{\mathcal {C}}}$ if and only if ${\mathbf {y}}\in \{ {\mathbf {x}} \} \oplus {\mathcal {C}}$.

#### *Remark 10*

It is clear that for any cone ${\mathcal {C}}$ the associated binary relation is translation invariant (i.e, if $\forall {\mathbf {u}} \in {\mathbb {R}}^m: ({\mathbf {x}},{\mathbf {y}}) \in R_{{\mathcal {C}}} \Rightarrow ({\mathbf {x}}+{\mathbf {u}}, {\mathbf {y}}+{\mathbf {u}}) \in R_{{\mathcal {C}}}$) and also multiplication invariant by any positive real (i.e., $\forall \alpha > 0: ({\mathbf {x}}, {\mathbf {y}}) \in R_{{\mathcal {C}}} \Rightarrow (\alpha {\mathbf {x}}, \alpha {\mathbf {y}}) \in R_{{\mathcal {C}}}$). Conversely, given a binary relation *R* which is translation invariant and multiplication invariant by any positive real, the set ${\mathcal {C}}_R := \{ {\mathbf {y}} - {\mathbf {x}}\, | \, ({\mathbf {x}}, {\mathbf {y}}) \in R \}$ is a cone. The above two operations are inverses of each other, i.e., to a cone ${\mathcal {C}}$ one associates a binary relation $R_{{\mathcal {C}}}$ which is translation invariant and multiplication invariant by any positive real, and the associated cone of $R_{{\mathcal {C}}}$ is ${\mathcal {C}}$, and conversely starting from a binary relation *R* which is translation invariant and multiplication invariant by any positive real one obtains the cone ${\mathcal {C}}_R$ and the binary relation associated to this cone is *R*. In short, there is a natural one to one correspondence between cones and translation invariant and multiplication-invariant-by-positive-reals binary relations on ${\mathbb {R}}^m$.

Note that for a positive multiplication invariant relation *R* the set ${\mathcal {C}}_R \, = \, \{ {\mathbf {y}} - {\mathbf {x}}\, | \, {\mathbf {x}}R{\mathbf {y}}\, \}$ is a cone. We restrict our attention to relations which are translation invariant as well in order to get the above mentioned bijection between cones and relations.

Also note if a positive multiplication invariant and translation invariant relation *R* is such that $\emptyset \ne R \ne {\mathbb {R}}^m\times {\mathbb {R}}^m$, then the associated cone ${\mathcal {C}}_R$ is non-trivial. Relations associated to non-trivial cones are non-empty and not equal to all of ${\mathbb {R}}^m\times {\mathbb {R}}^m$.

#### *Remark 11*

In general the binary relation $R_{{\mathcal {C}}}$ associated to a cone is not reflexive nor transitive nor anti-symmetric. For instance, the binary relation $R_{{\mathcal {C}}}$ is reflexive if and only if ${\mathbf {0}} \in {\mathcal {C}}$. The following definitions are needed in order to state for which cones the associated binary relation is anti-symmetric and/or transitive.

#### **Definition 12**

Pointed cone and convex cone. A cone ${\mathcal {C}}$ is pointed if and only if ${\mathcal {C}} \cap -{\mathcal {C}} \subseteq \{ {\mathbf {0}} \}$ where $-{\mathcal {C}} = \{ -c\ |\ c \in {\mathcal {C}} \}$ and ${\mathcal {C}}$ is convex if and only if $\forall c_1 \in {\mathcal {C}}, c_2 \in {\mathcal {C}}, \forall \alpha \text{ such } \text{ that } 0\le \alpha \le 1: \alpha c_1 + (1-\alpha ) c_2 \in {\mathcal {C}}$.

With these definitions we can specify for which cones the associated relation is transitive and/or anti-symmetric:

#### Proposition 3

*Let* ${\mathcal {C}}$ *be a cone and* $R_{{\mathcal {C}}}$ *its associated binary relation* (*i.e.*, $R_{{\mathcal {C}}}\, =\, \{({\mathbf {x}},{\mathbf {y}})\, |\, {\mathbf {y}} - {\mathbf {x}}\in {\mathcal {C}} \}$) . *Then the following statements hold.*

- $R_{{\mathcal {C}}}$ *is translation and positive multiplication invariant,*
- $R_{{\mathcal {C}}}$ *is anti-symmetric if and only if* ${\mathcal {C}}$ *is pointed*,
- $R_{{\mathcal {C}}}$ *is transitive if and only if* ${\mathcal {C}}$ *is convex, and moreover,*
- $R_{{\mathcal {C}}}$ *is reflexive if and only if* $\mathbf {{\mathbf {0}}} \in {\mathcal {C}}$.

A similar statement can be made if we go in the other direction, i.e.:

#### Proposition 4

*Let* *R* *be a translation and positive multiplication invariant binary relation and the* ${\mathcal {C}}_R$ *the associated cone* (*i.e.*, ${\mathcal {C}}_R \, =\, \{ {\mathbf {y}} - {\mathbf {x}} \, | \, ({\mathbf {x}},{\mathbf {y}}) \in R \}$). *Then the following statements hold.*

- $${\mathcal {C}}_R$$ *is a cone,*
- *R* *is anti-symmetric if and only if* ${\mathcal {C}}_R$ *is pointed,*
- *R* *is transitive if and only if* ${\mathcal {C}}_R$ *is convex, and moreover,*
- *R* *is reflexive if and only if* $\mathbf {{\mathbf {0}}} \in {\mathcal {C}}_R$.

In the following definition some important subsets in ${\mathbb {R}}^m, m \ge 1$ are introduced.

#### **Definition 13**

Let *m* be a natural number bigger or equal to 1. The non-negative orthant (aka hyperoctant) of ${\mathbb {R}}^m$, denoted by ${\mathbb {R}}_{\ge 0}^m$ is the set of all elements in ${\mathbb {R}}^m$ whose coordinates are non-negative. Furthermore, the zero-dominated orthant, denoted by ${\mathbb {R}}^m_{\succ {\mathbf {0}}}$, is the set ${\mathbb {R}}^m_{\ge 0} \setminus \{ {\mathbf {0}}\}$. Analogously we define the non-positive orthant of ${\mathbb {R}}^m$, denoted by ${\mathbb {R}}_{\le 0}$, as the set of elements in ${\mathbb {R}}^m$ the coordinates of which are non-positive. Furthermore, the set of elements in ${\mathbb {R}}^m$ which dominate the zero vector ${\mathbf {0}}$, denoted by ${\mathbb {R}}_{\prec {\mathbf {0}}}^m$, is the set ${\mathbb {R}}_{\le 0}^m \setminus \{{\mathbf {0}} \}$. The set of positive reals is denoted by ${\mathbb {R}}_{>0}$ and the set of non-negative reals is denoted by ${\mathbb {R}}_{\ge 0}$.

#### *Remark 12*

The sets defined in the previous definition are cones.

#### Proposition 5

*The Pareto order* $\prec _{Pareto}$ *on* ${\mathbb {R}}^m$ *is given by the cone order with cone* ${\mathbb {R}}^m_{\succ {\mathbf {0}}}$, *also referred to as the Pareto cone.*

#### *Remark 13*

As ${\mathbb {R}}^m_{\succ {\mathbf {0}}}$ is a pointed and convex cone, the associated binary relation is irreflexive, anti-symmetric and transitive (see Proposition 3). Of course, this can be verified more directly.

The reason to view the Pareto order as derived from a cone is that it gives the opportunity to study this order more geometrically. For instance, the definition and many of the properties of the very important hypervolume indicator (to be defined later) readily become intuitive. A reason for deviating from the Pareto cone could be to add constraints to the trade-off between solutions. Moreover, see later for a discussion, the more general cones turned out to be very useful in generalizing the hypervolume indicator and influence the distribution of points in the approximation set to the Pareto front.

Alternatives to the standard Pareto order on ${\mathbb {R}}^{m}$ can be easily imagined and constructed by using pointed, convex cones. The alternatives can be used, for instance, in preference articulation.

### Time complexity of basic operations on ordered sets

Partial orders do not have cycles. Let *R* be a partial order. It is easy to see that *R* does not have cycles. We show that the associated strict partial order does not have cycles. That is, there do not exist

$$\begin{aligned} (b_1,b_2) \in R\setminus \Delta , (b_2, b_3) \in R\setminus \Delta , \cdots , (b_{t-1},b_1) \in R\setminus \Delta \end{aligned}$$

where $\Delta$ is the diagonal. For suppose such $b_i, i=1, \cdots , t-1$ can be found with this property. Then by transitivity of $R\setminus \Delta$ (see Proposition 1), we get $(b_1, b_{t-1}) \in R\setminus \Delta$. By assumption, we have $(b_{t-1},b_1)\in R\setminus \Delta$. Again by transitivity, we get $(b_1, b_1) \in R\setminus \Delta$ which is a contradiction. In other words, *R* does not have cycles. (The essence of the above argument is, that any strict partial order does not have cycles.) The absence of cycles for (strict) partial orders gives rise to the following proposition.

#### Proposition 6

*Let* *S* *be a (strict) partially ordered set. Then any finite, non-empty subset of* *S* *has minimal elements (with respect to the partial order). In particular, any finite, non-empty subset* $Y \subset {\mathbb {R}}^m$ *has minimal elements with respect to the Pareto order* $\prec _{Pareto}$. *Also any, finite non-empty subset* $X \subset {\mathcal {X}}$ *has minimal elements with respect to* $\prec _{{\mathbf {f}}}$.

The question arises: How fast can the minimal elements be obtained?

#### Proposition 7

*Given a finite partially ordered set* $(X, \preceq )$, *the set of minimal elements can be obtained in time* $\varTheta (n^2)$.

#### *Proof*

A double nested loop can check non-domination for each element. For the lower bound consider the case that all elements in *X* are incomparable. Only in this case is *X* the minimal set. It requires time $\Omega (n^2)$ to compare all pairs (Daskalakis et al. 2011). $\square$

Fortunately, in case of the Pareto ordered set $(X, \prec _{Pareto})$, one can find the minimal set faster. The algorithm suggested by Kung et al. (1975) combines a dimension sweep algorithm with a divide and conquer algorithm and finds the minimal set in time $O(n (\log n))$ for $d=2$ and in time $O(n (\log n)^{d-2})$ for $d \ge 3$. Hence, in case of small finite decision spaces, efficient solutions can be identified without much effort. In the case of large combinatorial or continuous search spaces, however, optimization algorithms are needed to find them.

## Scalarization techniques

Classically, multiobjective optimization problems are often solved using scalarization techniques (see, for instance, Miettinen 2012). Also in the theory and practice of evolutionary multiobjective optimization scalarization plays an important role, especially in the so-called decomposition based approaches.

In brief, scalarization means that the objective functions are aggregated (or reformulated as constraints), and then a constrained single-objective problem is solved. By using different parameters of the constraints and aggregation function, it is possible to obtain different points on the Pareto front. However, when using such techniques, certain caveats have to be considered. In fact, one should always ask the following two questions:

1. Does the optimization of scalarized problems result in efficient points?
2. Can we obtain all efficient points or vectors on the Pareto front by changing the parameters of the scalarization function or constraints?

We will provide four representative examples of scalarization approaches and analyze whether they have these properties.

### Linear weighting

A simple means to scalarize a problem is to attach non-negative weights (at least one of them positive) to each objective function and then to minimize the weighted sum of objective functions. Hence, the multiobjective optimization problem is reformulated to:

#### **Definition 14**

Linear Scalarization Problem. The linear scalarization problem (LSP) of an MOP using a weight vector $w \in {\mathbb {R}}^m_{\succ {\mathbf {0}}}$, is given by

$$\begin{aligned} \text{ minimize } \sum _{i=1}^m w_i f_i(x), x\in {\mathcal {X}}. \end{aligned}$$

#### Proposition 8

*The solution of an LSP is on the Pareto front, no matter which weights in* ${\mathbb {R}}^m_{\succ {\mathbf {0}}}$ *are chosen.*

#### *Proof*

We show that the solution of the LSP cannot be a dominated point, and therefore, if it exists, it must necessarily be a non-dominated point. Consider a solution of the LSP against some weights $w \in {\mathbb {R}}^m_{\succ {\mathbf {0}}}$, say $x^*$ and suppose this minimal point is dominated. Then there exists an objective vector ${\mathbf {y}} \in {\mathbf {f}}({\mathcal {X}})$ with $\forall i \in \{1, \ldots , m\}\ \ y_i \le f_i(x^*)$ and for some index $j \in \{1, \ldots , m\}$ it holds that $y_j < f_j(x^*)$. Hence, it must also hold that $\sum _{i=1}^m w_i y_i < \sum _{i=1}^m w_i f_i(x^*)$, which contradicts the assumption that $x^*$ is minimal. $\square$

In the literature the notion of convexity (concavity) of Pareto fronts is for the most part not defined formally. Possible formal definitions for convexity and concavity are as follows.

#### **Definition 15**

Convex Pareto front. A Pareto front is convex if and only if ${\mathcal {Y}}_N \oplus {\mathbb {R}}^m_{\ge 0}$ is convex.

#### **Definition 16**

Concave Pareto front. A Pareto front is concave if and only if ${\mathcal {Y}}_N \oplus {\mathbb {R}}^m_{\le 0}$ is convex.

#### Proposition 9

*In case of a convex Pareto front, for each solution in* ${\mathcal {Y}}_N$ *there is a solution of a linear scalarization problem for some weight vector* $w \in {\mathbb {R}}^m_{\succ {\mathbf {0}}}$.

**Fig. 2:**
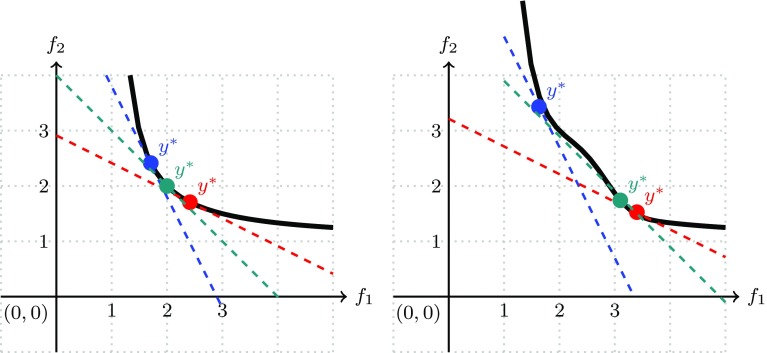
Linear scalarization problems with different weights for (1) convex Pareto fronts, and (2) concave Pareto fronts

If the Pareto front is non-convex, then, in general, there can be points on the Pareto front which are the solutions of no LSP. Practically speaking, in the case of concave Pareto fronts, the LSP will tend to give only extremal solutions, that is, solutions that are optimal in one of the objectives. This phenomenon is illustrated in Fig. 2, where the tangential points of the dashed lines indicate the solution obtained by minimizing an LSP for different weight choices (colors). In the case of the non-convex Pareto front (Fig. 2, right), even equal weights (dark green) cannot lead to a solution in the middle part of the Pareto front. Also, by solving a series of LSPs with minimizing different weighted aggregation functions, it is not possible to obtain this interesting part of the Pareto front.

#### **Chebychev scalarization**

Another means of scalarization, that will also uncover points in concave parts of the Pareto front, is to formulate the weighted Chebychev distance to a reference point as an objective function.

##### **Definition 17**

Chebychev Scalarization Problem. The Chebychev scalarization problem (CSP) of an MOP using a weight vector $\mathbf {\lambda } \in {\mathbb {R}}^m_{\succ {\mathbf {0}}}$, is given by

$$\begin{aligned} \text{ minimize } \max _{i \in \{1, \ldots , m\}} \lambda _i |f_i({\mathbf {x}}) - z^{*}_i|, {\mathbf {x}}\in {\mathcal {X}}, \end{aligned}$$

where ${\mathbf {z}}^{*}$ is a reference point, i.e., the ideal point defined as $z^{*}_i = \inf _{{\mathbf {x}} \in {\mathcal {X}}}f_i({\mathbf {x}})$ with $i=1,\cdots , m$.

##### Proposition 10

*Let us assume a given set of mutually non-dominated solutions in* ${\mathbb {R}}^m$ *(e.g., a Pareto front). Then for every non-dominated point* ${\mathbf {p}}$ *there exists a set of weights for a CSP, that makes this point a minimizer of the CSP provided the reference point* ${\mathbf {z}}^{*}$ *is properly chosen* (*i.e., the vector* ${\mathbf {p}}-{\mathbf {z}}^{*}$ *either lies in the positive or negative orthant*).

**Fig. 3:**
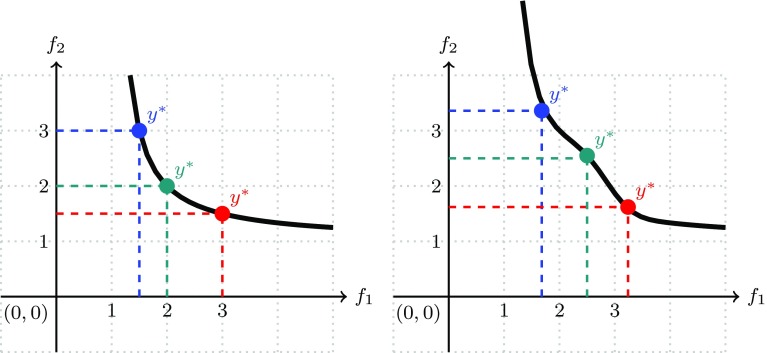
Chebychev scalarization problems with different weights for (1) convex Pareto fronts, and (2) concave Pareto fronts

Practically speaking, Proposition 10 ensures that by changing the weights, all points of the Pareto front can, in principle, occur as minimizers of CSP. For the two example Pareto fronts, the minimizers of the Chebychev scalarization function are points on the iso-height lines of the smallest CSP function value which still intersect with the Pareto front. Clearly, such points are potentially found in convex parts of Pareto fronts as illustrated in Fig. 3 (left) as well as in concave parts (right).However, it is easy to construct examples where a CSP obtains minimizers in weakly dominated points (see Definition 8). Think for instance of the case ${\mathbf {f}}({\mathcal {X}}) = [0,1]^2$. In this case all points on the line segment $\overline{(0,0)^{\top }, (0,1)^{\top }}$ and on the line segment $\overline{(0,0)^{\top } (1,0)^{\top }}$ are solutions of some Chebychev scalarization. (The ideal point is ${\mathbf {0}} = (0,0)^{\top }$, one can take as weights (0, 1) for the first scalarization, and (1, 0) for the second scalarization; the Pareto front is equal to $\{ (0,0)^{\top } \}$).

In order to prevent this, the augmented Chebychev scalarization provides a solution. It reads:

2 $$\begin{aligned} \text{ minimize } \max _{i \in \{1, \ldots , m\}} \lambda _i f_i({\mathbf {x}}) + \epsilon \sum _{i=1}^n f_i({\mathbf {x}}), {\mathbf {x}} \in {\mathcal {X}}, \end{aligned}$$

where $\epsilon$ is a sufficiently small, positive constant.

#### $\boldsymbol{\epsilon}$-constraint method

A rather straightforward approach to turn a multiobjective optimization problem into a constraint single-objective optimization problem is the $\epsilon$-constraint method.

##### **Definition 18**

$\epsilon$–constraint Scalarization. Given a MOP, the $\epsilon$–constraint scalarization is defined as follows. Given $m-1$ constants $\epsilon _1\in {\mathbb {R}}, \dots , \epsilon _{m-1}\in {\mathbb {R}}$,

$$\begin{aligned} \text{ minimize } f_1({\mathbf {x}}), \text{ subject } \text{ to } g_1({\mathbf {x}}) \le \epsilon _1, \dots , g_{m-1}({\mathbf {x}}) \le \epsilon _{m-1}, \end{aligned}$$

where $f_1,g_1,\dots , g_{m-1}$ constitute the *m* components of vector function ${\mathbf {f}}$ of the multiobjective optimization problem (see Definition 1).

**Fig. 4:**
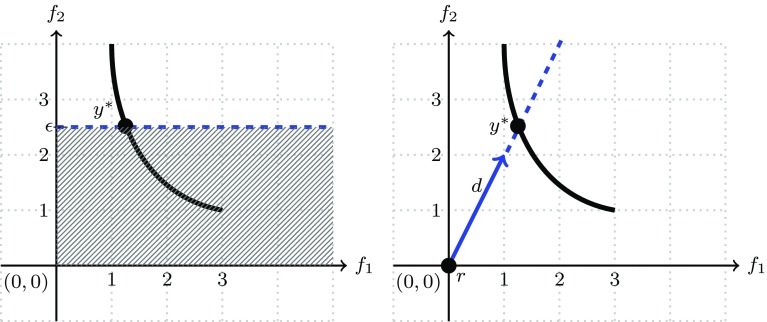
Re-formulation of multiobjective optimziation problems as single-objective constraint handling optimization problems

The method is illustrated in Fig. 4 (left) for $\epsilon _1=2.5$ for a biobjective problem. Again, by varying the constants $\epsilon _1\in {\mathbb {R}}, \dots , \epsilon _{m-1}\in {\mathbb {R}}$, one can obtain different points on the Pareto front. And again, among the solutions weakly dominated solutions may occur. It can, moreover, be difficult to choose an appropriate range for the $\epsilon$ values, if there is no prior knowledge of the location of the Pareto front in ${\mathbb {R}}^m$.

#### Boundary intersection methods

Another often suggested way to find an optimizer is to search for intersection points of rays with the attained subset ${\mathbf {f}}({\mathcal {X}})$ (Jaszkiewicz and Słowiński 1999). For this method, one needs to choose a reference point in ${\mathbb {R}}^m$, say ${\mathbf {r}}$, which, if possible, dominates all points in the Pareto front. Alternatively, in the Normal Boundary Intersection method (Das and Dennis 1998) the rays can emanate from a line (in the bi-objective case) or an $m-1$ dimensional hyperplane, in which case lines originate from different evenly spaced reference points (Das and Dennis 1998). Then the following problem is solved:

##### **Definition 19**

Boundary Intersection Problem. Let ${\mathbf {d}} \in {\mathbb {R}}^m_{\succ {\mathbf {0}}}$ denote a direction vector and ${\mathbf {r}} \in {\mathbb {R}}^m$ denote the reference vector. Then the boundary intersection problem is formulated as:

$$\begin{aligned} \text{ minimize } t, \\&\text{ subject } \text{ to } \\&(a)\ {\mathbf {r}} + t {\mathbf {d}} - {\mathbf {f}}({\mathbf {x}}) = 0, \\&(b)\ {\mathbf {x}} \in {\mathcal {X}}, \text{ and } \\&(c)\ t\in {\mathbb {R}}_{\ge 0} \end{aligned}$$

Constraints (a) and (b) in the above problem formulation enforce that the point is on the ray and also that there exists a pre-image of the point in the decision space. Because *t* is minimized, we obtain the point that is closest to the reference point in the direction of ${\mathbf {d}}$. This method allows some intuitive control on the position of resulting Pareto front points. Excepting rare degenerate cases, it will obtain points on the boundary of the attainable set ${\mathbf {f}}({\mathcal {X}})$. However, it also requires an approximate knowledge of the position of the Pareto front. Moreover, it might result in dominated points if the Pareto front is not convex. The method is illustrated in Fig. 4 (left) for a single direction and reference point.

## Numerical algorithms

Many of the numerical algorithms for solving multiobjective optimization problems make use of scalarization with varying parameters. It is then possible to use single-objective numerical optimization methods for finding different points on the Pareto front.

Besides these, there are methods that focus on solving the Karush-Kuhn-Tucker conditions. These methods aim for covering all solutions to the typically underdetermined nonlinear equation system given by these condition. Again, for the sake of clarity and brevity, in the following treatment, we will focus on the unconstrained case, noting that the full Karush-Kuhn-Tucker and Fritz-John conditions also feature equality and inequality constraints (Kuhn and Tucker 1951).

### **Definition 20**

Local Efficient Point. A point ${\mathbf {x}} \in {\mathcal {X}}$ is locally efficient, if there exists $\epsilon \in {\mathbb {R}}_{> 0}$ such that $\not \exists {\mathbf {y}} \in {\mathcal {B}}_\epsilon ({\mathbf {x}}): {\mathbf {y}} \prec _{{\mathbf {f}}} {\mathbf {x}} \text{ and } {\mathbf {x}} \ne {\mathbf {y}}$, where ${\mathcal {B}}_\epsilon ({\mathbf {x}})$ denotes the open $\epsilon$-ball around ${\mathbf {x}}$.

### **Theorem 1**

*Fritz–John Conditions. A neccessary condition for* ${\mathbf {x}} \in {\mathcal {X}}$ *to be locally efficient is given by*

$$\begin{aligned} \exists \lambda \succ {\mathbf {0}}: \sum _{i=1}^m \lambda _i \nabla f_i(\mathbf {{\mathbf {x}}}) = 0 \text{ and } \sum _{i=1}^m \lambda _i=1. \end{aligned}$$

### **Theorem 2**

*Karush–Kuhn–Tucker Conditions. A point* $\mathbf {{\mathbf {x}}} \in {\mathcal {X}}$ *is locally efficient, if it satisfies the Fritz–John conditions and for which all objective functions are convex in some open* $\epsilon$-*ball* ${\mathcal {B}}_\epsilon (\mathbf {{\mathbf {x}}})$ *around* $\mathbf {{\mathbf {x}}}$.

### *Remark 14*

The equation in the Fritz–John Condition typically does not result in a unique solution. For an *n*-dimensional decision space ${\mathcal {X}}$ we have $n+1$ equations and we have $m+n$ unknowns (including the $\lambda$ multipliers). Hence, in a non-degenerate case, the solution set is of dimension $m-1$.

It is possible to use continuation and homotopy methods to obtain all the solutions. The main idea of *continuation methods* is to find a single solution of the equation system and then to expand the solution set in the neighborhood of this solution. To decide in which direction to expand, it is necessary to maintain an archive, say *A*, of points that have already been obtained. To obtain a new point $\mathbf {{\mathbf {x}}}_{new}$ in the neighborhood of a given point from the archive $\mathbf {{\mathbf {x}}} \in A$ the homotopy method conducts the following steps:

1. Using the implicit function theorem a tangent space at the current point is obtained. It yielded an $m-1$ dimensional hyperplane that is tangential to ${{\mathbf {f}}}(\mathbf {{\mathbf {x}}})$ and used to obtain a predictor. See for the implicit function theorem, for instance, Krantz and Parks (2003).
2. A point on the hyperplane in the desired direction is obtained, thereby avoiding regions that are already well covered in *A*.
3. A corrector is computed minimizing the residual $||\sum \lambda _i f_i(\mathbf {{\mathbf {x}}})||$.
4. In case the corrector method succeeded to obtain a new point in the desired neighborhood, the new point is added to the archive. Otherwise, the direction is saved (to avoid trying it a second time).

See Hillermeier (2001) and Schütze et al. (2005) for examples and more detailed descriptions. The continuation and homotopy methods require the efficient set to be connected. Moreover, they require points to satisfy certain regularity conditions (local convexity and differentiability).

Global multiobjective optimization research is still a very active field of research. There are some promising directions, such as subdivision techniques (Dellnitz et al. 2005), Bayesian global optimization (Emmerich et al. 2016), and Lipschitz optimization (Žilinskas 2013). However, these require the decision space to be of low dimension.

Moreover, there is active research on derivative-free methods for numerical multiobjective optimization. Direct search techniques have been devised, for instance, Custódio et al. (2011), and by Audet et al. (2010). For a summary of derivative-free methods, see Custódio et al. (2012).

## Evolutionary multiobjective optimization

Evolutionary algorithms are a major branch of bio-inspired search heuristics, which originated in the 1960ties and are widely applied to solve combinatorial and non-convex numerical optimization problems. In short, they use paradigms from natural evolution, such as selection, recombination, and mutation to steer a population (set) of individuals (decision vectors) towards optimal or near-optimal solutions (Bäck 1996).

Multiobjective evolutionary algorithms (MOEAs) generalize this idea, and typically they are designed to gradually approach sets of Pareto optimal solutions that are well-distributed across the Pareto front. As there are—in general—no single-best solutions in multiobjective optimization, the selection schemes of such algorithms differ from those used in single-objective optimization. First MOEAs were developed in the 1990ties—see, e.g., Kursawe (1990) and Fonseca and Fleming (1993), but since around the year 2001, after the first book devoted exclusively to this topic was published by Deb (2001), the number of methods and results in this field grew rapidly.

With some exceptions, the distinction between different classes of evolutionary multiobjective optimization algorithms is mainly due to the differences in the paradigms used to define the selection operators, whereas the choice of the variation operators is generic and dependent on the problem. As an example, one might consider NSGA-II (see Deb et al. 2002) as a typical evolutionary multiobjective optimization algorithm; NSGA-II can be applied to continuous search spaces as well as to combinatorial search spaces. Whereas the selection operators stay the same, the variation operators (mutation, recombination) must be adapted to the representations of solutions in the decision space.

There are currently three main paradigms for MOEA designs. These are:

1. Pareto based MOEAs: The Pareto based MOEAs use a two-level ranking scheme. The Pareto dominance relation governs the first ranking and contributions of points to diversity is the principle of the second level ranking. The second level ranking applies to points that share the same position in the first ranking. NSGA-II (see Deb et al. 2002) and SPEA2 (see Zitzler and Thiele 1999) are two popular algorithms that fall into this category.
2. Indicator based MOEAs: These MOEAs are guided by an indicator that measures the performance of a set, for instance, the hypervolume indicator or the R2 indicator. The MOEAs are designed in a way that improvements concerning this indicator determine the selection procedure or the ranking of individuals.
3. Decomposition based MOEAs: Here, the algorithm decomposes the problem into several subproblems, each one of them targeting different parts of the Pareto front. For each subproblem, a different parametrization (or weighting) of a scalarization method is used. MOEA/D and NSGA-III are well-known methods in this domain.

In this tutorial, we will introduce typical algorithms for each of these paradigms: NSGA-II, SMS-EMOA, and MOEA/D. We will discuss important design choices, and how and why other, similar algorithms deviate in these choices.

### Pareto based algorithms: NSGA-II

The basic loop of NSGA-II (Deb et al. 2002) is given by Algorithm 1.

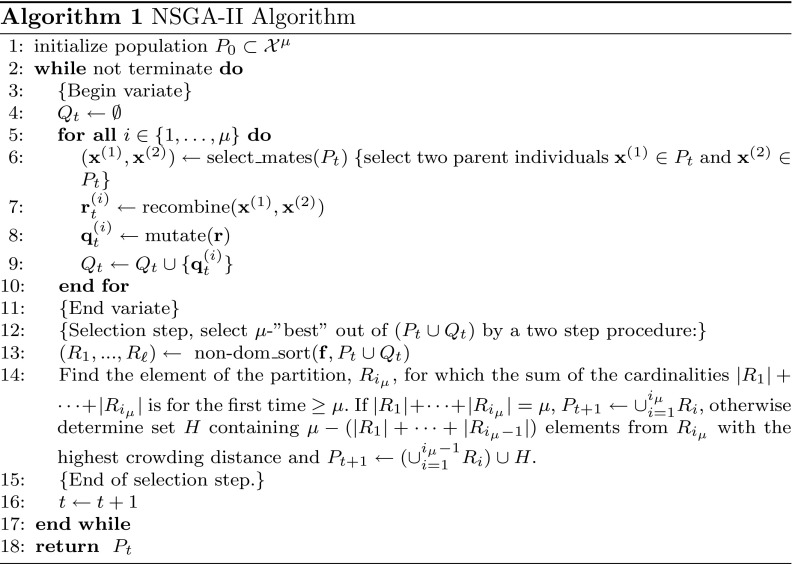



Firstly, a population of points is initialized. Then the following *generational loop* is repeated. This loop consists of two parts. In the first, the population undergoes a variation. In the second part, a selection takes place which results in the new generation-population. The generational loop repeats until it meets some termination criterion, which could be convergence detection criterion (cf. Wagner et al. 2009) or the exceedance of a maximal computational budget.

In the variation part of the loop $\lambda$ offspring are generated. For each offspring, two parents are selected. Each one of them is selected using binary tournament selection, that is drawing randomly two individuals from $P_t$ and selecting the better one concerning its rank in the population. The parents are then recombined using a standard recombination operator. For real-valued problems *simulated binary crossover* (SBX) is used (see Deb and Argawal 1995). Then the resulting individual is mutated. For real-valued problem polynomial mutation (PM) is used (see Mateo and Alberto 2012). This way, $\lambda$ individuals are created, which are all combinations or modifications of individuals in $P_t$. Then the parent and the offspring populations are merged into $P_t \cup Q_t$.

In the second part, the selection part, the $\mu$ best individuals of $P_t \cup Q_t$ with respect to a multiobjective ranking are selected as the new population $P_{t+1}$.

Next we digress in order to explain the multiobjective ranking which is used in NSGA-II. The key ingredient of NSGA-II that distinguishes it from genetic algorithms for single-objective optimization, is the way the individuals are ranked. The *ranking procedure of NSGA-II* consists of two levels. First, non-dominated sorting is performed. This ranking solely depends on the Pareto order and does not depend on diversity. Secondly, individuals which share the same rank after the first ranking are then ranked according to the crowding distance criterion which is a strong reflection of the diversity.

Let $\text {ND}(P)$ denote the non-dominated solutions in some population. Non-dominated sorting partitions the populations into subsets (layers) based on Pareto non-dominance and it can be specified through recursion as follows.

3 $$\begin{aligned} R_1= & {} \mathrm {ND}(P) \end{aligned}$$

4 $$\begin{aligned} R_{k+1}= & {} \mathrm {ND}(P \setminus \cup _{i=1}^k R_i), k = 1, 2, \dots \end{aligned}$$

**Fig. 5:**
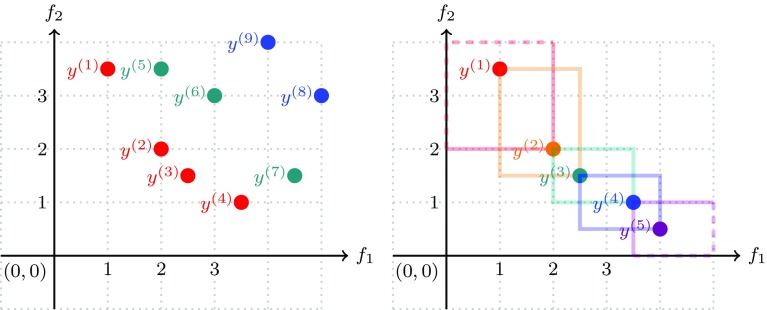
Illustration of non-dominated sorting (left) and crowding distance (right)

As in each step of the recursion at least one solution is removed from the population, the maximal number of layers is |*P*|. We will use the index $\ell$ to denote the highest non-empty layer. The rank of the solution after non-dominated sorting is given by the subindex *k* of $R_k$. It is clear that solutions in the same layer are mutually incomparable. The non-dominated sorting procedure is illustrated in Fig. 5 (upper left). The solutions are ranked as follows $R_1 = \{{\mathbf {y}}^{(1)}, {\mathbf {y}}^{(2)}, {\mathbf {y}}^{(3)}, {\mathbf {y}}^{(4)}\}$, $R_2 = \{{\mathbf {y}}^{(5)}, {\mathbf {y}}^{(6)}, {\mathbf {y}}^{(7)}\}$, $R_3 = \{{\mathbf {y}}^{(8)}, {\mathbf {y}}^{(9)}\}$.

Now, if there is more than one solution in a layer, say *R*, a secondary ranking procedure is used to rank solutions within that layer. This procedure applies the crowding distance criterion. The crowding distance of a solution ${\mathbf {x}} \in R$ is computed by a sum over contributions $c_i$ of the *i*-th objective function:

5 $$\begin{aligned} l_i({\mathbf {x}}):= & {} \max ( \{f_i({\mathbf {y}}) | {\mathbf {y}} \in R \setminus \{{\mathbf {x}}\} \wedge f_i({\mathbf {y}}) \le f_i({\mathbf {x}}) \} \cup \{ -\infty \}) \end{aligned}$$

6 $$\begin{aligned} u_i({\mathbf {x}}):= & {} \min ( \{f_i({\mathbf {y}}) | {\mathbf {y}} \in R \setminus \{{\mathbf {x}}\} \wedge f_i({\mathbf {y}}) \ge f_i({\mathbf {x}})\}\cup \{ \infty \}) \end{aligned}$$

7 $$\begin{aligned} c_i({\mathbf {x}}):= & {} u_i - l_i, \quad i=1, \dots , m \end{aligned}$$

The crowding distance is now given as:

8 $$\begin{aligned} c({\mathbf {x}}) := \frac{1}{m}\sum _{i=1}^m c_i({\mathbf {x}}), {\mathbf {x}}\in R \end{aligned}$$

For $m=2$ the crowding distances of a set of mutually non-dominated points are illustrated in Fig. 5 (upper right). In this particular case, they are proportional to the perimeter of a rectangle that just is intersecting with the neighboring points (up to a factor of $\frac{1}{4}$). Practically speaking, the value of $l_i$ is determined by the nearest neighbor of ${\mathbf {x}}$ to the left *according to the i-coordinate*, and $l_i$ is equal to the *i*-th coordinate of this nearest neighbor, similarly the value of $u_i$ is determined by the nearest neighbor of ${\mathbf {x}}$ to the right *according to the i-coordinate*, and $u_i$ is equal to the *i*-th coordinate of this right nearest neighbor. The more space there is around a solution, the higher is the crowding distance. Therefore, solutions with a high crowding distance should be ranked better than those with a low crowding distance in order to maintain diversity in the population. This way we establish a second order ranking. If the crowding distance is the same for two points, then it is randomly decided which point is ranked higher.

Now we explain the non-dom_sort procedure in line 13 of Algorithm 1 the role of *P* is taken over by $P_t \cap Q_t$: In order to select the $\mu$ best members of $P_t \cup Q_t$ according to the above described two level ranking, we proceed as follows. Create the partition $R_1, R_2, \cdots , R_{\ell }$ of $P_t \cup Q_t$ as described above. For this partition one finds the first index $i_{\mu }$ for which the sum of the cardinalities $|R_1|+\cdots +|R_{i_{\mu }}|$ is for the first time $\ge \mu$. If $|R_1|+\cdots +|R_{i_{\mu }}|=\mu$, then set $P_{t+1}$ to $\cup _{i=1}^{i_{\mu }}R_i$, otherwise determine the set *H* containing $\mu -(|R_1|+\cdots +|R_{i_{\mu }-1}|)$ elements from $R_{i_{\mu }}$ with the highest crowding distance and set the next generation-population, $P_{t+1}$, to $(\cup _{i=1}^{i_{\mu }-1}R_i)\cup H$.

Pareto-based Algorithms are probably the largest class of MOEAs. They have in common that they combine a ranking criterion based on Pareto dominance with a diversity based secondary ranking. Other common algorithms that belong to this class are as follows. The Multiobjective Genetic Algorithm (MOGA) (Fonseca and Fleming 1993), which was one of the first MOEAs. The PAES (Knowles and Corne 2000), which uses a grid partitioning of the objective space in order to make sure that certain regions of the objective space do not get too crowded. Within a single grid cell, only one solution is selected. The Strength Pareto Evolutionary Algorithm (SPEA) (Zitzler and Thiele 1999) uses a different criterion for ranking based on Pareto dominance. The strength of an individual depends on how many other individuals it dominates and by how many other individuals dominate it. Moreover, clustering serves as a secondary ranking criterion. Both operators have been refined in SPEA2 (Zitzler et al. 2001), and also it features a strategy to maintain an archive of non-dominated solutions. The Multiobjective Micro GA.

Coello and Pulido (2001) is an algorithm that uses a very small population size in conjunction with an archive. Finally, the Differential Evolution Multiobjective Optimization (DEMO) (Robic and Filipic 2005) algorithm combines concepts from Pareto-based MOEAs with a variation operator from differential evolution, which leads to improved efficiency and more precise results in particular for continuous problems.

### Indicator-based algorithms: SMS-EMOA

A second algorithm that we will discuss is a classical algorithm following the paradigm of indicator-based multiobjective optimization. In the context of MOEAs, by a performance indicator (or just indicator), we denote a scalar measure of the quality of a Pareto front approximation. Indicators can be *unary*, meaning that they yield an absolute measure of the quality of a Pareto front approximation. They are called *binary*, whenever they measure how much better one Pareto front approximation is concerning another Pareto front approximation.

The SMS-EMOA (Emmerich et al. 2005) uses the hypervolume indicator as a performance indicator. Theoretical analysis attests that this indicator has some favorable properties, as the maximization of it yields approximations of the Pareto front with points located on the Pareto front and well distributed across the Pareto front. The hypervolume indicator measures the size of the dominated space, bound from above by a reference point.

For an approximation set $A \subset {\mathbb {R}}^m$ it is defined as follows:

9 $$\begin{aligned} \text {HI(A)} = \mathrm {Vol}(\{{\mathbf {y}} \in {\mathbb {R}}^m: {\mathbf {y}} \preceq _{Pareto} {\mathbf {r}} \wedge \exists {\mathbf {a}} \in A: {\mathbf {a}} \preceq _{Pareto} {\mathbf {y}} \}) \end{aligned}$$

Here, $\text{ Vol }(.)$ denotes the Lebesgue measure of a set in dimension *m*. This is length for $m=1$, area for $m=2$, volume for $m=3$, and hypervolume for $m \ge 4$. Practically speaking, the hypervolume indicator of *A* measures the size of the space that is dominated by *A*. The closer points move to the Pareto front, and the more they distribute along the Pareto front, the more space gets dominated. As the size of the dominated space is infinite, it is necessary to bound it. For this reason, the reference point ${\mathbf {r}}$ is introduced.

The SMS-EMOA seeks to maximize the hypervolume indicator of a population which serves as an approximation set. This is achieved by considering the contribution of points to the hypervolume indicator in the selection procedure. Algorithm 2 describes the basic loop of the standard implementation of the SMS-EMOA.

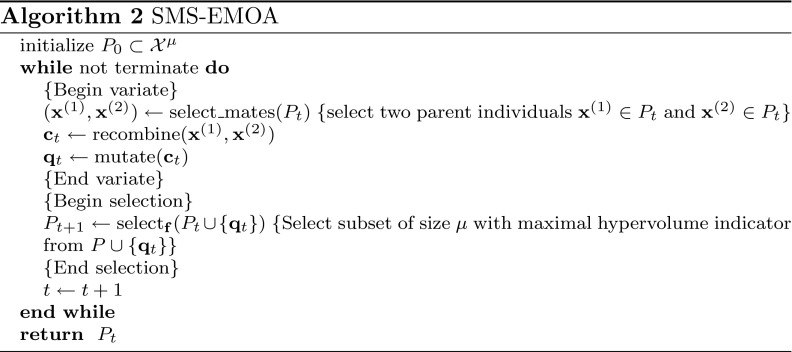



The algorithm starts with the initialization of a population in the search space. Then it creates only *one* offspring individual by recombination and mutation. This new individual enters the population, which has now size $\mu +1$. To reduce the population size again to the size of $\mu$, a subset of size $\mu$ with maximal hypervolume is selected. This way as long as the reference point for computing the hypervolume remains unchanged, the hypervolume indicator of $P_t$ can only grow or stay equal with an increasing number of iterations *t*.

Next, the details of the selection procedure will be discussed. If all solutions in $P_t$ are non-dominated, the selection of a subset of maximal hypervolume is equivalent to deleting the point with the smallest (exclusive) hypervolume contribution. The hypervolume contribution is defined as:

$$\begin{aligned} \Delta \mathrm {HI}({\mathbf {y}}, Y) = \mathrm {HI}(Y) - \mathrm {HI}(Y \setminus \{{\mathbf {y}}\}) \end{aligned}$$

**Fig. 6:**
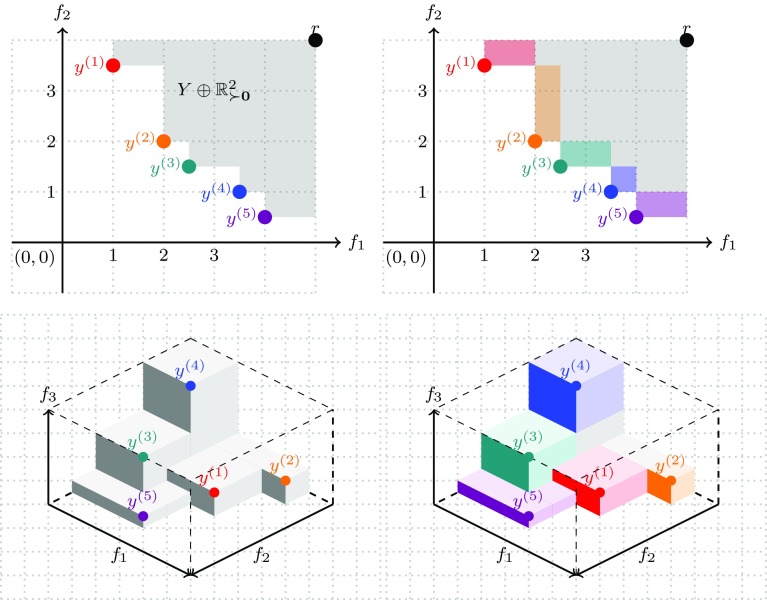
Illustration of 2-D hypervolume (top left), 2-d hypervolume contributions (top right), 3-D hypervolume (bottom left), and 3-D hypervolume contributions (bottom right)

An illustration of the hypervolume indicator and hypervolume contributions for $m=2$ and, respectively, $m=3$ is given in Fig. 6. Efficient computation of all hypervolume contributions of a population can be achieved in time $\varTheta (\mu \log \mu )$ for $m=2$ and $m=3$ (Emmerich and Fonseca 2011). For $m=3 \text{ or } 4$, fast implementations are described in Guerreiro and Fonseca (2017). Moreover, for fast logarithmic-time incremental updates for 2-D an algorithm is described in Hupkens and Emmerich (2013). For achieving logarithmic time updates in SMS-EMOA, the non-dominated sorting procedure was replaced by a procedure, that sorts dominated solutions based on age. For $m>2$, fast incremental updates of the hypervolume indicator and its contributions were proposed in for more than two dimensions (Guerreiro and Fonseca 2017).

In case dominated solutions appear the standard implementation of SMS-EMOA partitions the population into layers of equal dominance ranks, just like in NSGA-II. Subsequently, the solution with the smallest hypervolume contribution on the worst ranked layer gets discarded.

SMS-EMOA typically converges to regularly spaced Pareto front approximations. The density of these approximations depends on the local curvature of the Pareto front. For biobjective problems, it is highest at points where the slope is equal to $-45^{\circ }$ (Auger et al. 2009). It is possible to influence the distribution of the points in the approximation set by using a generalized cone-based hypervolume indicator. These indicators measure the hypervolume dominated by a cone-order of a given cone, and the resulting optimal distribution gets more uniform if the cones are acute, and more concentrated when using obtuse cones (see Emmerich et al. 2013).

Besides the SMS-EMOA, there are various other indicator-based MOEAs. Some of them also use the hypervolume indicator. The original idea to use the hypervolume indicator in an MOEA was proposed in the context of archiving methods for non-dominated points. Here the hypervolume indicator was used for keeping a bounded-size archive (Knowles et al. 2003). Besides, in an early work hypervolume-based selection which also introduced a novel mutation scheme, which was the focus of the paper (Huband et al. 2003). The term Indicator-based Evolutionary Algorithms (IBEA) (Zitzler and Künzli 2004) was introduced in a paper that proposed an algorithm design, in which the choice of indicators is generic. The hypervolume-based IBEA was discussed as one instance of this class. Its design is however different to SMS-EMOA and makes no specific use of the characteristics of the hypervolume indicator. The Hypervolume Estimation Algorithm (HypE) (Bader and Zitzler 2011) uses a Monte Carlo Estimation for the hypervolume in high dimensions and thus it can be used for optimization with a high number of objectives (so-called many-objective optimization problems). MO-CMA-ES (Igel et al. 2006) is another hypervolume-based MOEA. It uses the covariance-matrix adaptation in its mutation operator, which enables it to adapt its mutation distribution to the local curvature and scaling of the objective functions. Although the hypervolume indicator has been very prominent in IBEAs, there are some algorithms using other indicators, notably this is the R2 indicator (Trautmann et al. 2013), which features an ideal point as a reference point, and the averaged Hausdorff distance ($\Delta _p$ indicator) (Rudolph et al. 2016), which requires an aspiration set or estimation of the Pareto front which is dynamically updated and used as a reference. The idea of aspiration sets for indicators that require knowledge of the ‘true’ Pareto front also occurred in conjunction with the $\alpha$-indicator (Wagner et al. 2015), which generalizes the approximation ratio in numerical single-objective optimization. The Portfolio Selection Multiobjective Optimization Algorithm (POSEA) (Yevseyeva et al. 2014) uses the Sharpe Index from financial portfolio theory as an indicator, which applies the hypervolume indicator of singletons as a utility function and a definition of the covariances based on their overlap. The Sharpe index combines the cumulated performance of single individuals with the covariance information (related to diversity), and it has interesting theoretical properties.

### Decomposition-based algorithm: MOEA/D

Decomposition-based algorithms divide the problem into subproblems using scalarizations based on different weights. Each scalarization defines a subproblem. The subproblems are then solved simultaneously by dynamically assigning and re-assigning points to subproblems and exchanging information from solutions to neighboring sub-problems.

The method defines neighborhoods on the set of these subproblems based on the distances between their aggregation coefficient vectors. When optimizing a subproblem, information from neighboring subproblems can be exchanged, thereby increasing the efficiency of the search as compared to methods that independently solve the subproblems.

MOEA/D (Zhang and Li 2007) is a very commonly used decomposition based method, that succeeded a couple of preceding algorithms based on the idea of combining decomposition, scalarization and local search (Ishibuchi and Murata 1996; Jin et al. 2001; Jaszkiewicz 2002). Note that even the early proposed algorithms VEGA (Schaffer 1985) and the vector optimization approach of Kursawe (see Kursawe 1990) can be considered as rudimentary decomposition based approaches, where these algorithms obtain a problem decomposition by assigning different members of a population to different objective functions. These early algorithmic designs used subpopulations to solve different scalarized problems. In contrast, in MOEA/D one population with interacting neighboring individuals is applied, which reduces the complexity of the algorithm.

Typically, MOEA/D works with Chebychev scalarizations, but the authors also suggest other scalarization methods, namely scalarization based on linear weighting—which however has problems with approximating non-convex Pareto fronts—and scalarization based on boundary intersection methods—which requires additional parameters and might also obtain strictly dominated points.

MOEA/D evolves a population of individuals, each individual ${\mathbf {x}}^{(i)}\in P_t$ being associated with a weight vector $\mathbf {\lambda }^{(i)}$. The weight vectors $\mathbf {\lambda }^{(i)}$ are evenly distributed in the search space, e.g., for two objectives a possible choice is: $\lambda ^{(i)} = (\frac{\lambda -i}{\lambda },\frac{i}{\lambda })^{\top },\, i\, =\, 0, \ldots ,\mu$.

The *i*-th subproblem $g({\mathbf {x}}| \mathbf {\lambda }^{i}, {\mathbf {z}}^*)$ is defined by the Chebychev scalarization function (see also Eq. 2):

10 $$\begin{aligned} g({\mathbf {x}} | \mathbf {\lambda }^{(i)}, {\mathbf {z}}^*) = \max _{j \in \{1, \ldots , m\}}\{ \mathbf {\lambda }^{(i)}_j |f_j({\mathbf {x}}) - z^{*}_j|\} + \epsilon \sum _{j=1}^m\left( f_j({\mathbf {x}}) - z^{*}_j \right) \end{aligned}$$

The main idea is that in the creation of a new candidate solution for the *i*-th individual the neighbors of this individual are considered. A neighbor is an incumbent solution of a subproblem with similar weight vectors. The neighborhood of *i*-th individual is the set of *k* subproblems, for so predefined constant *k*, that is closest to $\mathbf {\lambda }^{(i)}$ in the Euclidean distance, including the *i*-th subproblem itself. It is denoted with *B*(*i*). Given these preliminaries, the MOEA/D algorithm—using Chebychev scalarization— reads as described in Algorithm 3.

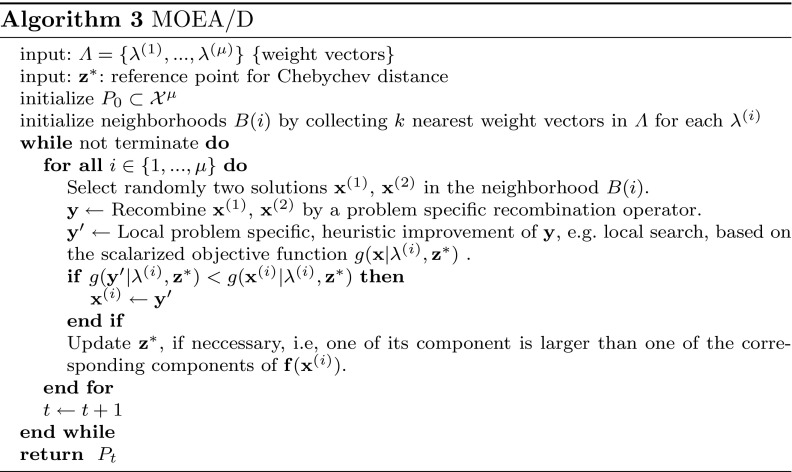



Note the following two remarks about MOEA/D: (1) Many parts of the algorithm are kept generic. Here, generic options are recombination, typically instantiated by standard recombination operators from genetic algorithms, and local improvement heuristic. The local improvement heuristic should find a solution in the vicinity of a given solution that does not violate constraints and has a relatively good performance concerning the objective function values. (2) MOEA/D has additional statements to collect all non-dominated solutions it generates during a run in an *external archive*. Because this external archive is only used in the final output and does not influence the search dynamics, it can be seen as a generic feature of the algorithm. In principle, an external archive can be used in all EMOAs and could therefore also be done in SMS-EMOA and NSGA-II. To make comparisons to NSGA-II and SMS-EMOA easier, we omitted the archiving strategy in the description.

Recently, decomposition-based MOEAs became very popular, also because they scale well to problems with many objective functions. The NSGA-III (Deb and Jain 2014) algorithm is specially designed for many-objective optimization and uses a set of reference points that is dynamically updated in its decomposition. Another decomposition based technique is called Generalized Decomposition (Giagkiozis et al. 2014). It uses a mathematical programming solver to compute updates, and it was shown to perform well on continuous problems. The combination of mathematical programming and decomposition techniques is also explored in other, more novel, hybrid techniques, such as Directed Search (Schütze et al. 2016), which utilizes the Jacobian matrix of the vector-valued objective function (or approximations to it) to find promising directions in the search space, based on desired directions in the objective space.

## Performance assessment

In order to make a judgement (that is, gain insight into the advantages and disadvantages) of multiobjective evolutionary (or for that matter also deterministic) optimizers we need to take into account (1) the computational resources used, and (2) the quality of the produced result(s).

The current state of the art of multiobjective optimization approaches are mainly compared empirically though theoretical analyses exist (see, for instance, the convergence results described in Rudolph and Agapie (2000),indicators with respect Beume et al. (2011) albeit for rather simple problems as more realistic problems elude mathematical analysis.

The most commonly computational resource which is taken into account is the computation time which is very often measured implicitly by counting fitness function evaluations—in this respect, there is no difference with single-objective optimization. In contrast to single-objective optimization, in multiobjective optimization, a close distance to a (Pareto) optimal solution is not the only thing required but also good coverage of the entire Pareto front. As the results of multiobjective optimization algorithms are (finite) approximation sets to the Pareto front we need to be able to say when one Pareto front approximation is better than another. One good way to define when one approximation set is better than another is as in Definition 22 (see Zitzler et al. 2003).

### **Definition 21**

Approximation Set of a Pareto Front. A finite subset *A* of ${\mathbb {R}}^m$ is an approximation set of a Pareto front if and only if *A* consists of mutually (Pareto) non-dominated points.

### **Definition 22**

Comparing Approximation Sets of a Pareto Front. Let $A \text{ and }$ *B* be approximation sets of a Pareto front in ${\mathbb {R}}^m$. We say that *A* is better than *B* if and only if every $b \in B$ is weakly dominated by at least one element $a \in A$ and $A \ne B$. Notation: $A \rhd B$.

**Fig. 7:**
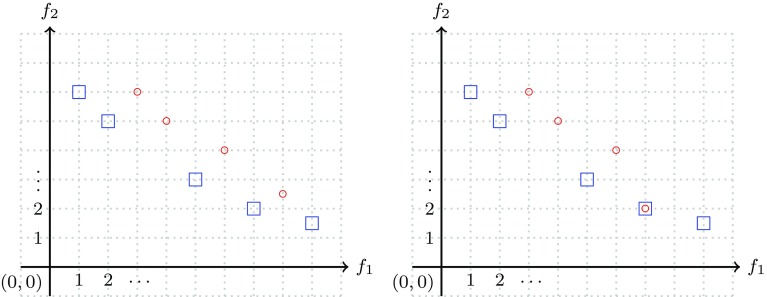
In the left picture, the set of points denoted by blue squares is better than ($\rhd$) the set consisting of the red-circle points. Also in the right picture the set consisting of blue squares is better than the set of red-circle points—in this case the intersection of the two sets is non-empty

**Fig. 8:**
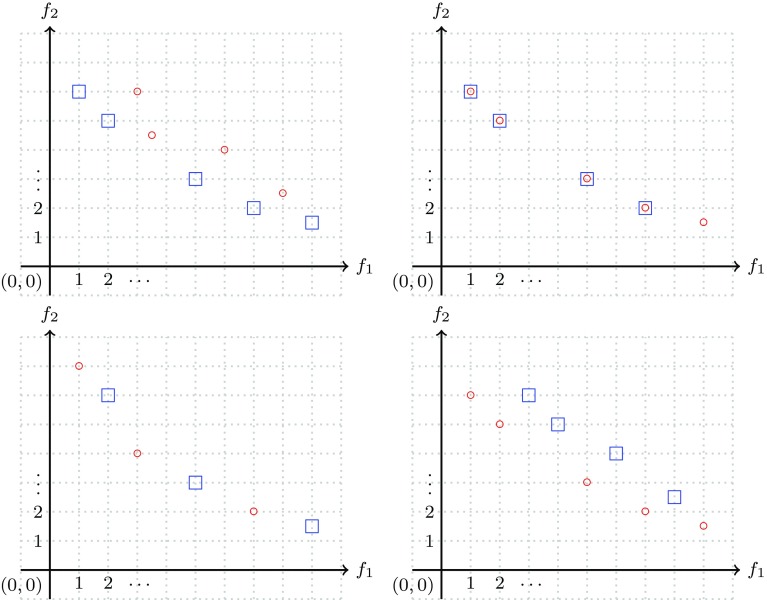
In each of the pictures, the set consisting of the blue square points is *not* better than the set consisting of the red circle points. Clearly, in each of the two pictures on the right the set consisting of the red circle points is better than the set consisting of the blue square points

In Fig. 7 examples are given of the case of one set being better than another and in Fig. 8 examples are given of the case that a set is not better than another.

This relation on sets has been used in Zitzler et al. (2003) to classify performance indicators for Pareto fronts. To do so, they introduced the notion of completeness and compatibility of these indicators with respect to the set relation ‘is better than’.

### **Definition 23**

Unary Set Indicator. A unary set indicator is a mapping from finite subsets of the objective space to the set of real numbers. It is used to compare (finite) approximations to the Pareto front.

### **Definition 24**

Compatibility of Unary Set Indicators concerning the ‘is better than’ order on Approximation Sets. A unary set indicator *I* is compatible concerning the ‘is better than’ or $\rhd$-relation if and only if $I(A) > I(B) \Rightarrow A \rhd B$. A unary set indicator *I* is complete with respect to the ‘is better than’ or $\rhd$-relation if and only if $A \rhd B \Rightarrow I(A) > I(B)$. If in the last definition we replace > by $\ge$ then the indicator is called weakly-complete.

The hypervolume indicator and some of its variations are complete. Other indicators compared in the paper (Zitzler et al. 2003) are weakly-complete or not even weakly-complete. It has been proven in the same paper that no unary indicator exists that is complete and compatible at the same time. Moreover for the hypervolume indicator $\text{ HI }$ it has be shown that $\text{ HI } (A) > \text{ HI } (B) \Rightarrow \lnot (B \rhd A)$. The latter we call weakly-compatible.

In all the discussions of the hypervolume indicator, the assumption is that all points of the approximation sets under consideration dominate the reference point. Recently, variations of the hypervolume indicator have been proposed—the so-called free hypervolume indicators—that do not require the definition of a reference point and are complete and weakly-compatible for all approximation sets (Zitzler et al. 2003).

Besides unary indicators, one has introduced binary indicators (see Riquelme et al. 2015). The most used ones are unary indicators followed up by binary indicators. For binary indicators, the input is a pair of approximation sets and the output is again a real number. Here the goal is to determine which of the two approximation sets is the better one (and how much better it is)^1^. Binary indicators can also be used, if the true Pareto front is known, e.g., in benchmarking on test problems. A common binary indicator is the binary $\epsilon$-indicator. In order to introduce this indicator we first define for each $\delta \in {\mathbb {R}}$ a binary relation on the points in ${\mathbb {R}}^m$.

### **Definition 25**

$\delta$-domination. Let $\delta \in {\mathbb {R}}$ and let $a \in {\mathbb {R}}^m$ and $b \in {\mathbb {R}}^m$. We say that *a* $\delta$-dominates *b* (notation: $a \preceq _{\delta } b$) if and only if $a_i \le b_i + \delta , i=1,\dots , m$.

Next, we can define the binary indicator $I_{\epsilon }$.

### **Definition 26**

The Binary Indicator $I_{\epsilon }$. Given two approximation sets *A* and *B*, then $I_{\epsilon }(A,B) := \inf _{\delta \in {\mathbb {R}}} \{ \forall b \in B \ \exists a \in A \text{ such } \text{ that } a \preceq _{\delta } b \}$.

Clearly for a fixed *B* the smaller $I_{\epsilon }(A,B)$ is the better the approximation set *A* is relative to *B*. The following properties hold: $A \rhd B \Rightarrow I_{\epsilon }(B,A) > 0$, the second notable property is as follows: $I_{\epsilon }(A,B) \le 0 \text{ and } I_{\epsilon }(B, A) > 0 \Rightarrow A \rhd B$. These two properties show that based on the binary $\epsilon$-indicator it is possible to decide whether or not *A* is better than *B*. In contrast, the knowledge of the hypervolume indicator on the sets *A* and *B* does not allow to decide whether or not *A* is better than *B*.

Some of indicators are useful in case there is knowledge or complete knowledge about the Pareto front. For instance (see Rudolph et al. 2016), it has been suggested to compute the Hausdorff distance (or variations of it) of an approximation set to the Pareto front. Moreover, the binary $\epsilon$-indicator can be transformed into a complete unary indicator in case the second input is the known Pareto front—note that this indicator needs to be minimized.

**Fig. 9:**
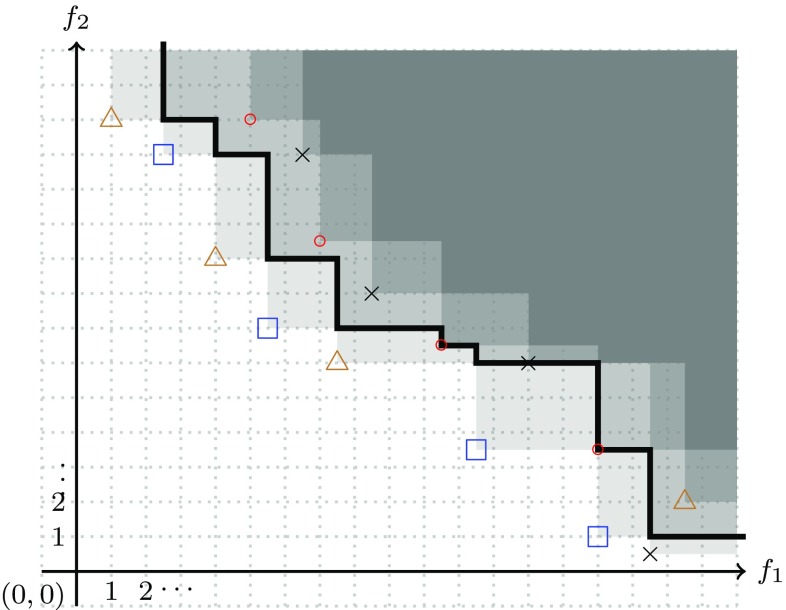
The median attainment curve for the case of four approximation sets; one approximation set consists of the blue squares, the second set consists of points denoted by brown triangles, the third consists of the red circles, and the fourth consists of points denoted by black crosses; the darker gray the region is the more approximation sets dominate it. The median attainment curve is the black polygonal line

Another useful way to learn about the behavior of evolutionary multiobjective algorithms is the attainment curve approach (see da Fonseca et al. 2001). The idea is to generalize the cumulative distribution function and for the study of algorithms it is approximated empirically. The distribution is defined on the set of (finite) approximation sets of the Pareto front. For each point in the objective space ${\mathbb {R}}^m$ it is the probability that the Pareto front approximation attains this point (that is, it is either one point in the approximation set, or it is dominated by some point in the approximation set). Formally, it reads

$$\begin{aligned} P(a^{(1)} \le z \vee a^{(2)} \le z \vee \dots \vee a^{(k)}\le z), \end{aligned}$$

where $A = \{ a^{(1)}, a^{(2)}, \dots , a^{(k)} \}$ is the approximation set and $z \in {\mathbb {R}}^m$. In other words *P* is the probability of an algorithm to find at least one solution which attains the goal *z* in a single run. We define the attainment function $\alpha _A : {\mathbb {R}}^m \rightarrow [0,1]$ associated to the approximation set *A* as follows:

$$\begin{aligned} \alpha _{A}(z) := P(a^{(1)} \le z \vee a^{(2)}\le z \vee \dots \vee a^{(k)}\le z). \end{aligned}$$

This function can be approximated by

$$\begin{aligned} \alpha _s(z) := \frac{1}{s}\sum _{i=1}^s {\mathcal {I}}(A_i, z), \end{aligned}$$

where $$A_1, \dots , A_s$$ are the outcome approximation sets of an algorithm in *s* runs of the algorithm and $${\mathcal {I}}: (\text{ set } \text{ of } \text{ approximation } \text{ sets }) \times {\mathbb {R}}^m \rightarrow \{0, 1 \}$$ is a function which associates to an approximation set and a vector in $${\mathbb {R}}^m$$ the value 1 in case the vector is a member of the approximation set or if some element of the approximation set dominates it, otherwise the value is 0. For $$m = 2 \text{ or } 3$$ we can plot the boundaries where this function changes its value. These are the attainment curves ($$m=2)$$ and attainment surfaces ($$m=3$$). In particular the median attainment curve/surface gives a good summary of the behavior of the optimizer. It is the boundary where the function changes from a level below 0.5 to a level higher than 0.5. Alternatively one can look at lower and higher levels than 0.5 in order to get an optimistic or respectively a pessimistic assessment of the performance.

In Fig. 9 an example of the median attainment curve is shown. We assume that the four approximation sets are provided by some algorithm.

## Recent topics in multiobjective optimization

Recently, there are many new developments in the field of multiobjective optimization. Next we will list some of the most important trends.

### Many-objective optimization

Optimization with more than 3 objectives is currently termed many-objective optimization [see, for instance, the survey (Li et al. 2015)]. This is to stress the challenges one meets when dealing with more than 3 objectives. The main reasons are:

1. problems with many objectives have a Pareto front which cannot be visualized in conventional 2D or 3D plots instead other approaches to deal with this are needed;
2. the computation time for many indicators and selection schemes become computationally hard, for instance, time complexity of the hypervolume indicator computation grows super-polynomially with the number of objectives, under the assumption that $P \ne NP$;
3. last but not least the ratio of non-dominated points tends to increase rapidly with the number of objectives. For instance, the probability that a point is non-dominated in a uniformly distributed set of sample points grows exponentially fast towards 1 with the number of objectives.

In the field of many-objective optimization different techniques are used to deal with these challenges. For the first challenge, various visualization techniques are used such as projection to lower dimensional spaces or parallel coordinate diagrams. In practice, one can, if the dimension is only slightly bigger than 3, express the coordinate values by colors and shape in 3D plots.

Naturally, in many-objective optimization indicators which scale well with the number of objectives (say polynomially) are very much desired. Moreover, decomposition based approaches are typically preferred to indicator based approaches.

The last problem requires, however, more radical deviations from standard approaches. In many cases, the reduction of the search space achieved by reducing it to the efficient set is not sufficiently adequate to allow for subsequent decision making because too many alternatives remain. In such cases, a stricter order than the Pareto order is required which requires additional preference knowledge. To elicit preference knowledge, interactive methods often come to the rescue. Moreover, in some cases, objectives are correlated which allows for grouping of objectives, and in turn, such groups can be aggregated to a single objective. Dimensionality reduction and community detection techniques have been proposed for identifying meaningful aggregation of objective functions.

### Preference modeling

The Pareto order is the most applied order in multiobjective optimization. However, different ranking schemes and partial orders have been proposed in the literature for various reasons. Often additional knowledge of user preferences is integrated. For instance, One distinguishes preference modeling according to at what stage of the optimization the preference information is collected (a priori, interactively, and a posteriori). Secondly one can distinguish the type of information requested from the decision maker, for instance, constraints on the trade-offs, relative importance of the objectives, and preferred regions on the Pareto front. Another way to elicit preference information is by ordinal regression; here the user is asked for pairwise comparisons of some of the solutions. From this data, the weights of utility functions are learned (Branke et al. 2015). The interested reader is also referred to interesting work on non-monotonic utility functions, using the Choquet integral (Branke et al. 2016). Notably, the topic of preference elicitation is one of the main topics in Multiple Criteria Decision Analysis (MCDA). In recent years collaboration between MCDA and multiobjective optimization (MOO) brought forward many new useful approaches. A recommended reference for MCDA is Belton and Stewart (2002). For a comprehensive overview of preference modelling in multiobjective optimization we refer to Li et al. (2017) and Hakanen et al. (2016). Moreover Greco et al. (2016) contains an updated collection of state of the art surveys on MCDA. A good reference discussing the integration of MCDA into MOO is Branke et al. (2008).

### Optimization with costly function evaluations

In industrial optimization very often the evaluation of (an) objective function(s) is achieved by simulation or experiments. Such evaluations are typically time-consuming and expensive. Examples of such costly evaluations occur in the optimization based on crash tests of automobiles, chemical experiments, computational fluid dynamics simulations, and finite element computations of mechanical structures. To deal with such problems techniques that need only a limited number of function evaluations have been devised. A common approach is to learn a surrogate model of the objective functions by using all available past evaluations. This is called surrogate model assisted optimization. One common approach is to optimize on the surrogate model to predict promising locations for evaluation and use these evaluations to further improve the model. In such methods, it is also important to add points for developing the model in under-explored regions of the search space. Some criteria such as expected improvement take both exploitation and exploration into account. Secondly, surrogate models can be used in pre-processing in the selection phase of evolutionary algorithms. To save time, less interesting points can be discarded before they would be evaluated by the costly and precise evaluator. Typically regression methods are used to construct surrogate models; Gaussian processes and neural networks are standard choices. Surrogate modeling has in the last decade been generalized to multiobjective optimization in various ways. Some important early work in this field was on surrogate assisted MOEAs (Emmerich et al. 2006) and ParEGO algorithm (Knowles 2006). A state of the art review can be found in Allmendinger et al. (2017).

### New bio-inspired paradigms

Inspiration by nature has been a creative force for dealing with optimization algorithm design. Apart from biological evolution, many other natural phenomena have been considered. While many of these algorithmic ideas have so far remained in a somewhat experimental and immature state, some non-evolutionary bio-inspired optimization algorithms have gained maturation and competitive performance. Among others, this seems to hold for particle swarm optimization (Reyes-Sierra and Coello Coello 2006), ant colony optimization (Barán and Schaerer 2003), and artificial immune systems Coello Coello and Cortés (2005). As with evolutionary algorithms, also these algorithms have first been developed for single-objective optimization, and subsequently, they have been generalized to multiobjective optimization. Moreover, there is some recent research on bio-inspired techniques that are specifically developed for multiobjective optimization. An example of such a development is the Predator-Prey Evolutionary Algorithm, where different objectives are represented by different types of predators to which the prey (solutions) have to adapt (Laumanns et al. 1998; Grimme and Schmitt 2006).

In the field of natural computing, it is also investigated whether algorithms can serve as models for nature. It is an interesting new research direction to view aspects of natural evolution as a multiobjective optimization process, and first such models have been explored in Rueffler (2006) and Sterck et al. (2011).

### Set-oriented numerical optimization

Traditionally, numerical techniques for multiobjective optimization are single point techniques: They construct a Pareto front by formulating a series of single-objective optimization problems (with different weights or constraints) or by expanding a Pareto front from a single solution point by point using continuation. In contrast, set-oriented numerical multiobjective optimization operates on the level of solution sets, the points of which are simultaneously improved, and that converge to a well-distributed set on the Pareto front. Only very recently such methods have been developed, and techniques that originated from evolutionary multiobjective optimization have been transferred into deterministic methods. A notable example is the hypervolume indicator gradient ascent method for multiobjective optimization (HIGA-MO) (Wang et al. 2017). In this method a set of say $\mu$ points is represented as a single vector of dimension $\mu n$, where *n* is the dimension of the search space. In real-valued decision space the mapping HI: ${\mathbb {R}}^{\mu d} \rightarrow {\mathbb {R}}$ from the such population vectors to the hypervolume indicator has a well-defined derivative in almost all points. The computation of such derivatives has been described in Emmerich and Deutz (2014). Viewing the vector of partial derivatives as a gradient, conventional gradient methods can be used. It requires, however, some specific adaptations in order to construct robust and practically usable methods for local optimization. On convex problems, fast linear convergence can be achieved. By using second-order derivatives in a hypervolume-based Newton-Raphson method, even quadratic convergence speed has been demonstrated empirically on a set of convex bi-objective problems. The theory of such second-order methods is subject to ongoing research (Hernández et al. 2014).

### Advanced performance assessment

Despite significant progress on the topic of performance assessment in recent years, there are still many unanswered questions. A bigger field of research is on performance assessment of interactive and many objective optimization. Moreover, the dependency of performance measures on parameters, such as the reference point of the hypervolume indicator requires further investigation. Some promising work in that direction was recently provided in Ishibuchi et al. (2017).

## How to get started?

In the following, we list some of the main resources for the field of (Evolutionary) Multiobjective Optimization.

- Introductory Books:
  - Jürgen Branke, Kalyanmoy Deb, Kaisa Miettinen, Roman Slowiński *Multiobjective Optimization : Interactive and evolutionary approaches*, Springer, 2008
  - Carlos Coello Coello et al. * Evolutionary Algorithms for Solving Multi-Objective Problems*, 2007, Springer
  - Kalyanmoy Deb *Multi-Objective Optimization using Evolutionary Algorithms*, Wiley, 2001
  - Matthias Ehrgott *Multicriteria Optimization*, Springer, 2005
  - Joshua Knowles, David Corne, Kalyanmoy Deb *Multiobjective Problem Solving from Nature*, Springer, 2007
  - Kaisa Miettinen *Multiobjective Nonlinear Optimization*, Kluwer, 2012
- Websites:
  - EMOO Repository by Carlos Coello Coello http://neo.lcc.uma.es/emoo/
  - SIMCO Open Problems http://simco.gforge.inria.fr/doku.php?id=openproblems; a collection of open problems and theoretical results on indicator based approaches and complexity theory.
  There are many implementations of multiobjective optimization algorithms available. Table 1 provides a table of **MOO Software**, including also some packages that include deterministic solvers.
- Conferences and Journals:
  - Conferences:
    - Conference on Evolutionary Computation (CEC), annual, published by IEEE
    - Evolutionary Multi-criterion Optimization (EMO) biannual conference, proceedings published by Springer LNCS
    - EVOLVE—a Bridge between Probability, Set Oriented Numerics and Evolutionary Computation, annual until 2015, published by Springer Studies in Computational Intelligence, continued as NEO see below
    - GECCO with EMO track, annual, published by ACM
    - Global Optimization Workshop (GO), biannual, published by diverse publishers (as special issues, and post-proceedings)
    - MCDM with EMO track, biannual, published by MCDM International Society
    - Numerical and Evolutionary Optimization(NEO), annual, published by Springer Advances in Computational Intelligence
    - and others
  - Journals^2^: COAP, ECJ, EJOR, IEEE TEVC, JOGO, MCDA Journal, and other Optimization, and Operations Research journals.

Aside from the resources mentioned above, there are many research groups and labs which maintain a repository of software accompanying their published research, e.g., the MODA group at Leiden University http://moda.liacs.nl and the research group of Carlos Fonseca at Coimbra University eden.dei.uc.pt/cmfonsec/software.html.

**Table 1:** Table of (evolutionary) multiobjective optimization software

Libraries (evolutionary) multiobjective optimization			
Name	Scope	Prog. Lang.	url or ref
*Public domain*			
ecr	EA and EMO	R	Bossek (2017)
JMetal	Metatheuristics/EMO	Java	Barba-González et al. (2017)
Liger	MOO/Design Optim.	C++	Giagkiozis et al. (2013)
MOEA framework	EMO	Java	moeaframework.org/
Opt4J	EMO	Java	opt4j.sourceforge.net
PISA	EMO	C++	Bleuler et al. (2003)
PyMOO	EMO	Python	www.openhub.net/p/pymoo
RODEOlib	Robust Optimization	Matlab	sourceforge.net/projects/rodeolib/
Shark Library	Machine Learning	C#	image.diku.dk/shark/
SUMO	Bayesian Optimization	Matlab	sumo.intec.ugent.be/SUMO
TEA	classical EA and MOO	C++	Emmerich and Hosenberg (2000)
vOptSolver	(Linear) MOO	Julia	voptsolver.github.io/vOptSolver
*Commercial software*			
EASY	Design Optimization	C++	velos0.ltt.mech.ntua.gr/EASY/
IND-NIMBUS	Design Optimization	N/A	ind-nimbus.it.jyu.fi
ISight	Design Optimization	N/A	www.simuleon.com
MODEfrontier	Design Optimization	N/A	www.esteco.com
Optimus	Design Optimization	N/A	www.noesissolutions.com
WWW-NIMBUS	Design Optimization	N/A	Miettinen and Mäkelä (2000)
*Performance assessment*			
Performance assessment test problems			
BBOB/COCO	Benchmarking Tool	C++	coco.gforge.inria.fr/
WFG	Test Suite	C++	www.wfg.csse.uwa.edu.au/toolkit/
ZDT/DTLZ	Test Suite	C++	esa.github.io/pagmo2/
Performance assessment software			
Attainment surfaces		R/C	lopez-ibanez.eu/eaftools
Hypervolume computation		C	lopez-ibanez.eu/hypervolume
Hypervolume computation	Link Collection	Various	ls11-www.cs.tu-dortmund.de/rudolph/hypervolume/start

## Summary and outlook

In this tutorial, we gave an introduction to the field of multiobjective optimization. We covered the topics of order-theoretical foundations, scalarization approaches, and optimality conditions. As solution methods, we discussed homotopy and evolutionary methods. In the context of Evolutionary methods, we discussed three state-of-the-art techniques in detail, namely NSGA-II, SMS-EMOA, and MOEA/D, each representing a key paradigm in evolutionary multiobjective algorithm design. NSGA-II served as a representative of Pareto based approaches, SMS-EMOA as an example of Indicator-based approaches, and MOEA/D as an example of decomposition based approaches. These algorithms have some advantages and disadvantages:

- Pareto-based approaches follow a straightforward design principle, that is directly based on Pareto dominance and diversity preservation (using, for instance, crowding distance). Usually, these algorithms require only a few parameters, and larger numbers of objective functions do not cause problems. However, it might be difficult to guarantee and measure convergence and achieve a very regular spacing of solutions.
- Indicator-based approaches use an indicator for the performance of an approximation set to guide the search. It is possible to assess their convergence behavior online, and they hold the promise to be more amenable to theoretical analysis. However, the computation time often increases rapidly with the number of dimensions and the distribution of points in the approximation sets might depend critically on the settings of the reference point or other parameters of the indicator.
- Decomposition-based methods provide a very flexible framework for algorithm design, as they can incorporate various scalarization methods. A disadvantage is that they require some a priori knowledge of the position of the Pareto front in the objective space and the number of weight vectors might grow exponentially with the objective space size, even if the Pareto front is of low dimension.

According to the above, choosing the right method depends much on the dimension of the objective space, the number of solutions one wants to output, the desired distribution of the solutions (knee-point focused or uniformly spread) and the a priori knowledge on the location and shape of the Pareto front.

Due to space constraints, many advanced topics in multiobjective optimization are not covered in depth. We refer for these topics to the literature. For instance, constraint handling (Coello Coello 2013), multimodality (Kerschke et al. 2016), non-convex global optimization (Žilinskas 2013), and combinatorial optimization (Ehrgott and Gandibleux 2000).

Multiobjective Optimization is a very active field of research. There are still many open, challenging problems in the area. For future development of the research field it will be essential to provide EMO algorithms that are built around a robust notion of performance and, ideally, also can be analyzed more rigorously. Major topics for current research are also uncertainty handling and robustness, many-objective optimization, theoretical foundations and computational complexity, generalizations, for instance, level set approximation, diversity optimization, and set-oriented optimization, customization and integration into multidisciplinary workflows, and scalability to big data, or expensive evaluations.

## Footnotes

1 Conceivably one can can introduce *k*-ary indicators. To our knowledge, so far they have not been used in multiobjective optimization.

2 a selection of journals in which many articles are published on (Evolutionary) Multiobjective Optimization.
